# Geographic differences in the prevalence of hypertension in Uganda: Results of a national epidemiological study

**DOI:** 10.1371/journal.pone.0201001

**Published:** 2018-08-01

**Authors:** Joseph Lunyera, Bruce Kirenga, John W. Stanifer, Samuel Kasozi, Thys van der Molen, Wenceslaus Katagira, Moses R. Kamya, Robert Kalyesubula

**Affiliations:** 1 Division of General Internal Medicine, Duke University School of Medicine, Durham, NC, United States of America; 2 Department of Medicine, Makerere University College of Health Sciences, Kampala, Uganda; 3 Makerere University Lung Institute, Kampala, Uganda; 4 Duke Global Health Institute, Duke University, Durham, NC, United States of America; 5 Department of General Practice and Elderly Care, and Groningen Research Institute for Asthma and COPD (GRIAC), University of Groningen, University Medical Center Groningen (UMCG), The Netherlands; 6 Department of Physiology, Makerere University College of Health Sciences, Kampala, Uganda; KEMRI-Wellcome Trust Research Program, KENYA

## Abstract

**Background:**

Hypertension accounts for more than 212 million global disability-adjusted life-years, and more than 15 million in sub-Saharan Africa. Identifying factors underlying the escalating burden of hypertension in sub-Saharan Africa may inform delivery of targeted public health interventions.

**Methods:**

As part of the cross-sectional nationally representative Uganda National Asthma Survey conducted in 2016, we measured blood pressure (BP) in the general population across five regions of Uganda. We defined hypertension as systolic BP ≥140 mmHg and/or diastolic BP ≥90 mmHg, or on-going use of medications for the purpose of lowering BP among adults (≥18 years of age); pre-hypertension as systolic BP between 120 and 140 mmHg and/or diastolic BP bteween 80 and 90 mmHg among adolescents and adults (≥12 years of age).

**Findings:**

Of 3416 participants who met inclusion criteria, 38.9% were male, and mean age ± SD was 33.8 ± 16.9 years. The age- and sex-adjusted prevalence of hypertension was 31.5% (95% confidence interval [CI] 30.2 to 32.8). The adjusted prevalence of hypertension was highest in the Central Region (34.3%; 95% CI 32.6 to 36.0), and it was comparable to that in the West and East Regions. However, compared with the Central Region, hypertension was significantly less prevalent in the North (22.0%; 95 CI 19.4 to 24.6) and West Nile Regions (24.1%; 95% CI 22.0 to 26.3). Adjustment for demographic characteristics (occupation, monthly income, and educational attainment) of participants did not account for the significantly lower prevalence of hypertension in the North and West Nile Regions. The prevalence of pre-hypertension was 38.8% (95% CI 37.7 to 39.8), and it was highly prevalent among young adults (21–40 years of age: 42.8%; 95% CI 41.2–44.5%) in all regions.

**Conclusions:**

Hypertension is starkly prevalent in Uganda, and numerous more people, including young adults are at increased risk. The burden of hypertension is highest in the Central, Western, and Eastern regions of the country; demographic characteristics did not fully account for the disparate regional burden of hypertension. Future studies should explore the potential additional impact of epidemiological shifts, including diet and lifestyle changes, on the development of hypertension.

## Introduction

Hypertension is the leading contributor to the global burden of disease (GBD). In the GBD Study of 2015, it accounted for more than 212 million global disability-adjusted life-years (DALYs)–reflecting a 40% increase from 1990 [1,2]. In sub-Saharan Africa, the corresponding change in DALYs attributable to hypertension ranged from 60% to 100% [1]. Further, with the increasing prevalence of hypertension in many sub-Saharan African countries [3], experts project a major increase in the burden of disease that will be attributable to hypertension in this setting [4,5]. Globally, factors driving the increase in the attributable DALYs for hypertension include demographic (e.g., population ageing, population growth, etc) and epidemiologic (e.g., adoption of western diets and lifestyles) transitions [2]. Of greater concern, however, is the paucity of data describing the role of these factors in the upward trajectory of the burden of hypertension in many sub-Saharan African countries.

Likewise, the prevalence of hypertension in Uganda appears to be high, ranging from 11% to 32%. Recent studies have highlighted inequities in the prevalence of hypertension among specific demographic groups, including men, middle-aged adults, professionals, and residents of the Central (home to the highly urbanized capital city of Kampala) and Eastern regions [6–12]. However, although prior studies have reported disparities in the prevalence of hypertension across geographic regions in the country [6–8], none examined whether correlates of the demographic transition, including educational attainment, occupation, and monthly income, explain these inequities. Therefore, as part of the Uganda National Asthma Survey (UNAS), we estimated the prevalence of hypertension in five regions of Uganda, and we sought to examine the potential impact of demographic factors on the prevalence of hypertension across geographic regions in Uganda.

## Methods

### Study population

The UNAS was a nationally representative cross-sectional study conducted from August to November 2016 among community-dwelling individuals, 12–100 years of age, recruited using a 3-stage cluster sampling strategy. In the first stage, we purposively sampled one district from each of the five regions of Uganda: Kampala (the capital and largest city) in the Central, Kiruhura in the West, Maracha in West Nile, Pader in the North, and Iganga in the East. Second, we sampled clusters from each selected district based on probability proportionate to size. Finally, we selected households from each cluster using simple random sampling from a household list generated by village leaders in each cluster. Adolescent and adult (≥12 years of age) inhabitants of selected households were eligible to participate in the study. Individuals who were residents of congregation settings (schools, prisons, homes), temporary residents (less than 2 weeks in household of selected villages), or those who did not provide written consent were excluded.

Mulago Hospital Research and Ethics committee and the Uganda National Council for Science and Technology approved the UNAS. Participants provided written informed consent and had liberty to terminate study participation at any time during the study. Children ≤18 years of age provided assent and the parent or legal guardian provided consent on their behalf.

### Definition of hypertension

We measured blood pressure (BP) using an Omron automated sphygmomanometer model HEM-907, which has an adjustable cuff size. Participants assumed a resting seated posture ≥10 minutes prior to two sequential BP readings taken 10 minutes apart. We considered the average of the two BP readings as the individual’s BP, and we estimated mean arterial pressure (MAP) as the sum of one-third of systolic BP and two-thirds of diastolic BP. We defined hypertension as systolic BP ≥140 mmHg and/or diastolic BP ≥90 mmHg, or self-reported on-going use of medications for the purpose of lowering BP; pre-hypertension as systolic BP between 120 and 140 mmHg and/or diastolic BP bteween 80 and 90 mmHg. Although the UNAS recruited both adolescents and adults, we defined hypertension among adults (≥18 years of age) only because the epidemiology of hypertension in children differs from that in adults.^13^ However, we defined prehypertension in both adolescents [13] and adults.

### Assessment of covariates

We designated urban-rural status at the cluster level based on the 2014 National Census report. Participants self-reported socio-demographic characteristics: age, sex, educational attainment, occupation, monthly income, smoking status, and alcohol consumption. Categories of educational attainment included less than primary school, completed primary school, and completed secondary school or higher. Subgroups for occupation comprised working in office settings (professionals and clerical jobs), peasant farming, construction work, unemployed, or other (not specified). Cigarette smoking included self-reported current or former smoking history. Alcohol consumption comprised self-reported history of alcohol drinking in the 12 months preceding study visit. Anthropometric measurements included height (measured without shoes to the nearest 0.1-centimeter using a stadiometer [SECA; Hamburg, Germany]) and weight (measured without shoes and in light clothing to the nearest 0.1 kilogram using a calibrated beam scale). We defined body mass index in kilograms per meter-squared.

### Statistical analysis

We computed survey weights for the UNAS to account for the differential probability of sampling households–described in detail in the **S1 File**. Continuous covariates were described using mean ± standard deviation (SD) or median (interquartile range) and categorical covariates were summarized as frequency and proportion. We compared covariates by geographical region using Mann-Whitney nonparametric test for continuous variables and Chi-square tests for categorical variables. We estimated the weighted prevalence of hypertension with 95% confidence interval (CI), and compared the prevalence of hypertension across regional and demographic strata using survey-weighted logistic regression.

We employed logistic regression models, accounting for sampling design effect, including clustering, using UNAS survey weights, to estimate the impact of demographic factors (age, sex, educational attainment, occupation, and monthly income) on the prevalence of hypertension across geographic regions. Variables were sequentially added to the model based on *a priori* assumptions [8]. The single-equation link test assessed for specification errors in the final model [14], and the uncentered variance inflation factors test evaluated collinearity. Subsequently, we generated predicted probabilities of hypertension and prehypertension by geographic region with values of covariates from the above logistic models fixed at the cohort mean or reference group.

Statistical significance tests are 2-sided at the 5% α level. All statistical analyses were conducted using STATA v.15 software (STATA corp., College Station, TX), and Arc Global Information Systems, ArcMap v10.4.1 (Environmental Systems Research Institute, Redlands, CA) was used to map the prevalence of hypertension across regions.

## Results

### Characteristics of study participants

Of 4310 individuals who were eligible, 3416 (79.3%) participated in the UNAS study–all of whom were included in the present analysis. Overall, 1327 (38.9%) participants were male, mean age ± SD was 33.8 ± 16.9 years, few completed secondary school or higher (10.7%), and the majority were peasant farmers (43.3%). Participants in the Central (vs. the overall study cohort) were less likely to be males (26.6% vs. 38.9%), but more likely to have completed ≥primary school (48.8% vs. 30.6%). They were also more likely to be occupied as a market vendor (8.5% vs. 3.3%) and to have greater monthly income (median income, per 1000 Ugandan Shillings 150 vs. 40).

Overall, mean ± SD systolic and diastolic BP were 124 ± 20 and 80 ± 14 mmHg, respectively. Mean arterial pressure was 95 ± 15 mmHg. Mean systolic BP was highest in the East (129 mmHg) and Central (127 mmHg); diastolic BP was highest in the West (84 mmHg) and Central (83 mmHg). Mean ± SD BMI was 21.3 ± 6.4 kg/m^2^; mean BMI was highest in the Central (24.4 kg/m^2^) and West (22.4 kg/m^2^) (**Table 1**).

**Table 1:** Socio-demographic characteristics of participants (≥12 years of age) in the Uganda National Asthma survey, stratified by geographic region.

Demographic characteristics	Overall N = 3416	Geographic region				
		Central	West	West Nile	North	East
Male sex, no. (%)	1327 (38.9)	207 (26.6)	360 (49.0)	271 (40.9)	263 (42.0)	226 (36.9)
Age, mean ± SD, y	33.8 ± 16.9	32.4 ± 14.8	37.6 ± 16.8	32.7 ± 16.1	29.0 ± 16.4	37.3 ± 19.1
Educational attainment, no. (%)						
<Primary school	2007 (58.8)	209 (26.8)	422 (57.4)	504 (76.0)	477 (76.2)	395 (64.4)
Completed primary school	1045 (30.6)	380 (48.8)	222 (30.2)	134 (20.2)	134 (21.4)	175 (28.6)
≥Secondary school^1^	364 (10.7)	190 (24.4)	91 (12.4)	25 (3.8)	15 (2.4)	43 (7.0)
Occupation, no. (%)						
Office setting^2^	151 (4.4)	83 (10.7)	25 (3.4)	12 (1.8)	15 (2.4)	16 (2.6)
Peasant farming	1476 (43.3)	7 (0.9)	435 (59.4)	398 (60.0)	346 (55.5)	290 (47.4)
Market vendor	111 (3.3)	66 (8.5)	20 (2.7)	11 (1.7)	3 (0.5)	11 (1.8)
Unemployed	560 (16.4)	223 (28.6)	31 (4.2)	131 (19.8)	27 (4.3)	148 (24.2)
Others^3^	1113 (32.6)	400 (51.4)	222 (30.3)	111 (16.7)	233 (37.3)	147 (24.0)
Monthly income, median [IQR]^4^	40 [20–100]	150 [60–300]	60 [30–100]	20 [10–48]	10 [5–30]	40 [20–90]
Cigarette smoking, no. (%)	242 (7.1)	28 (3.6)	60 (8.2)	107 (16.1)	33 (5.3)	14 (2.3)
Alcohol intake, no. (%)	621 (18.2)	195 (25.0)	57 (7.8)	233 (35.1)	108 (17.3)	28 (4.6)
Blood pressure, mean ± SD, mm Hg						
Systolic	124 ± 20	127 ± 19	123 ± 17	124 ± 19	121 ± 18	129 ± 24
Diastolic	80 ± 14	83 ± 13	84 ± 19	78 ± 11	77 ± 11	80 ± 13
Mean arterial pressure	95 ± 15	97 ± 14	97 ± 16	93 ± 13	91 ± 12	96 ± 16
Body mass index, mean ± SD, kg/m^2^	21.3 ± 6.4	24.4 ± 5.8	22.4 ± 5.4	18.5 ± 3.0	18.6 ± 3.1	20.8 ± 8.9

^1^Completed secondary ± tertiary ± university

^2^Includes professional and clerical jobs

^3^Examples include student, small business owner

^4^Per 1000 Uganda shillings

### Unadjusted prevalence of hypertension

The unadjusted national prevalence of hypertension among adults was 31.5% (95% CI: 30.3 to 32.8). The prevalence was higher among males (vs. females): 34.2% vs. 30.2% (P = 0.007); among individuals with <primary (vs. ≥primary) school: 35.3% vs. 28.1% (P = 0.014); and with increasing age (P for trend = 0.005) and monthly income (P for trend <0.001). The prevalence did not differ by rural-urban status: 32.0% (urban) vs. 30.9% (rural); P = 0.309. Although the there was no evidence for a trend in the prevalence of hypertension by occupation (P for trend = 0.379), the prevalence of hypertension was highest among market vendors (43.8%; 95% CI 38.8 to 49.0) (**Table 2**).

**Table 2:** Prevalence of hypertension among adults (≥18 years of age) in Uganda in 2016, stratified by socio-demographic characteristics.

Characteristics		Prevalence of hypertension		
		Prevalence (95% CI)	Prevalence ratio (95% CI)	P trend
Total population, n = 2794		31.5 (30.3–32.8)	NA	
Sex	Male	34.2 (32.3–36.2)	reference	0.01
	Female	30.2 (28.7–31.7)	0.83 (0.75–0.92)	
Age, years	18–20	12.8 (10.7–15.2)	reference	0.01
	21–40	22.9 (21.5–24.4)	2.03 (1.63–2.53)	
	41–60	49.9 (46.8–53.0)	6.80 (5.45–8.48)	
	>60	56.3 (51.8–60.7)	8.79 (6.64–11.62)	
Region	Central	32.0 (30.3–33.7)	reference	0.20
	West	36.1 (33.7–38.5)	1.20 (1.05–1.37)	
	West Nile	25.1 (22.9–27.4)	0.71 (0.62–0.82)	
	North	21.7 (19.2–24.4)	0.59 (0.50–0.70)	
	East	37.3 (34.9–39.8)	1.26 (1.11–1.44)	
Urban-rural status	Urban	32.0 (30.3–33.7)	reference	0.31
	Rural	30.9 (29.1–32.8)	0.95 (0.85–1.07)	
Educational attainment	<Primary school	35.3 (33.2–37.5)	reference	0.01
	Primary school	28.1 (26.4–29.9)	0.71 (0.63–0.81)	
	≥Secondary school^1^	29.9 (27.2–32.9)	0.78 (0.66–0.92)	
Occupation	Office setting^2^	30.6 (26.6–35.0)	reference	0.38
	Peasant farming	30.3 (28.4–32.2)	0.98 (0.79–1.22)	
	Market vendor	43.8 (38.8–49.0)	1.76 (1.31–2.37)	
	Unemployed	31.7 (29.3–34.3)	1.05 (0.84–1.32)	
	Others^3^	30.4 (28.3–32.5)	0.99 (0.79–1.23)	
Monthly income, UgX^4^	<20000	27.9 (25.1–30.9)	reference	<0.01
	20000–100000	31.8 (29.7–34.0)	1.21 (1.03–1.42)	
	100001–200000	34.9 (31.9–37.9)	1.38 (1.14–1.69)	
	≥200001	36.4 (33.6–39.4)	1.48 (1.23–1.78)	

CI, confidence interval; NA, not applicable; UgX, Ugandan Shillings (1 USD = 3,500 UgX)

^1^Completed secondary ± tertiary ± university

^2^Includes professional and clerical jobs

^3^Examples include student, small business owner

^4^Per 1000 Uganda shillings

**Fig 1:**
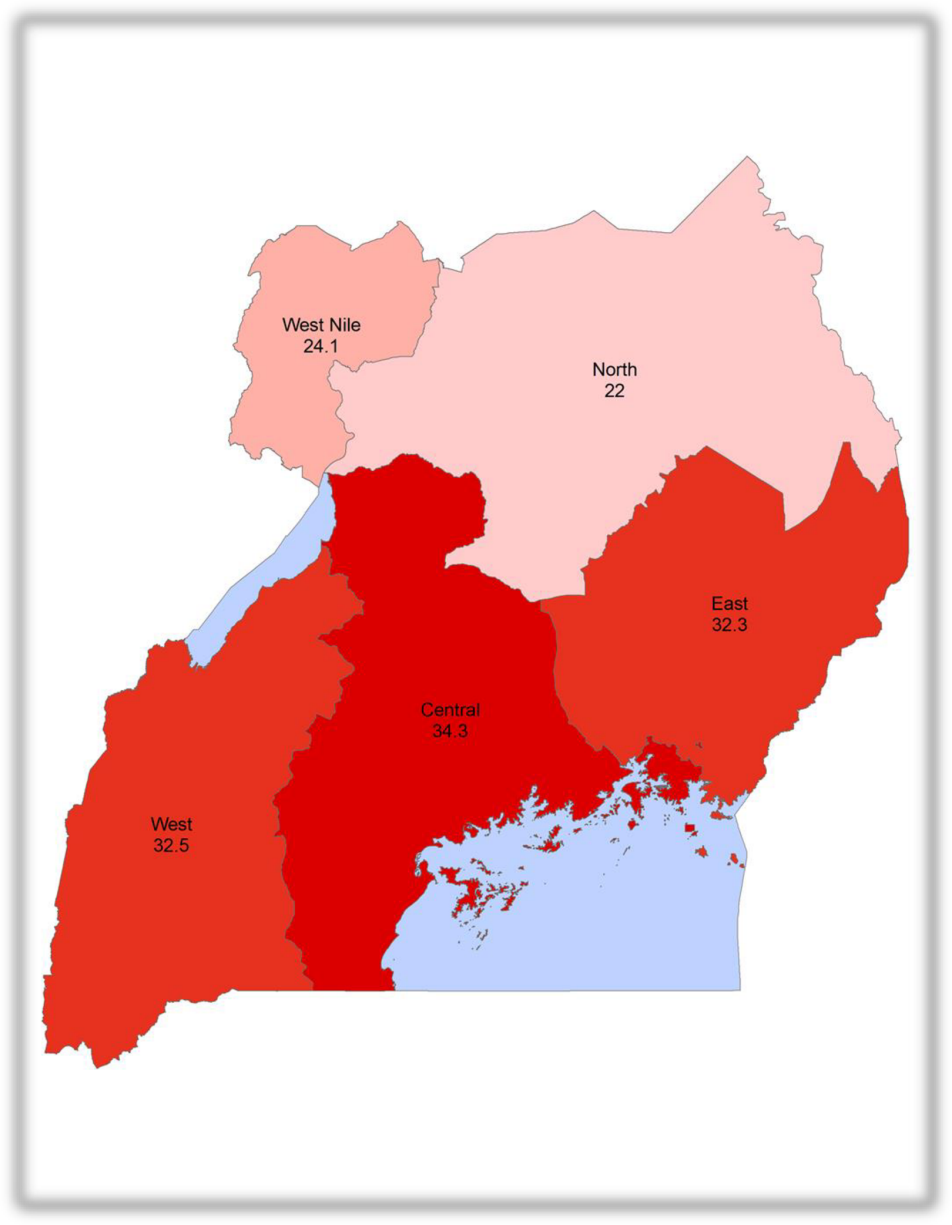
Age- and sex-adjusted prevalence of hypertension among adults (≥18 years of age) in Uganda by geographical region.

Prior to age- and sex-adjustment, hypertension was highly prevalent in the East (37.3%; 95% CI 34.9 to 39.8), West (36.1%; 95% CI 33.7 to 38.5), and Central (32.0%; 95% CI 30.3 to 33.7) Regions, but it was comparatively less prevalent in the North (21.7%; 95% CI 19.2 to 24.4) and West-Nile (25.1%; 95% CI 22.9 to 27.4) Regions (**Fig 1**). In unadjusted regression models with the Central Region as the reference, the prevalence of hypertension was 20% higher in the West (PR 1.20; 95% CI 1.05 to 1.37), 26% higher in the East (PR 1.26; 95% CI 1.11 to 1.44), 41% lower in the North (PR 0.59; 95% CI 0.50 to 0.70), and 29% lower in the West Nile (PR 0.71; 95% CI 0.62 to 0.82) Regions (**Table 2**).

### Adjusted prevalence of hypertension

Overall, the age- and sex-adjusted prevalence of hypertension among adults was 31.5% (95% CI 30.2–32.8). Adjustment for demographics annulled the excess prevalence of hypertension in the West and East, compared to the Central: P = 0.845 and P = 0.406 for West and East, respectively. As such, the age- and sex-adjusted prevalence of hypertension in the West (32.5%) and East (32.3%) were similar to that of the Central (34.3%). On the contrary, adjustment for demographics did not account for the low prevalence of hypertension in the North and West Nile Regions compared to the Central Region (all P remained <0.05). Thus, the age-and sex-adjusted prevalence of hypertension in the North (22.0%) and West Nile (24.1%) remained substantially lower than in the Central region (**Tables 3 and 4**).

**Table 3:** Association of region with prevalent hypertension among adults (≥18 years of age) before and after sequential adjustments for demographics and socio-economic status.

	Region	PR (95% CI)	P Value
Model 1: unadjusted	Central	Reference	NA
	West	1.20 (1.05–1.37)	0.02
	West Nile	0.71 (0.62–0.82)	<0.01
	North	0.59 (0.50–0.70)	<0.01
	East	1.26 (1.11–1.44)	0.01
Model 2: adjusted for age and sex	Central	Reference	NA
	West	0.92 (0.79–1.06)	0.17
	West Nile	0.58 (0.49–0.67)	<0.01
	North	0.50 (0.42–0.61)	<0.01
	East	0.90 (0.78–1.04)	0.11
Model 3: model 2 + SES	Central	Reference	NA
	West	1.02 (0.83–1.25)	0.85
	West Nile	0.72 (0.56–0.91)	0.02
	North	0.59 (0.45–0.78)	0.01
	East	1.08 (0.86–1.36)	0.41

PR, prevalence ratio; CI, confidence interval; NA, not applicable; SES, socio-economic status: urban-rural status, occupation, monthly income, and educational attainment

**Table 4:** Age- and sex-adjusted prevalence of pre-hypertension and hypertension by geographic region and age category.

	Pre-hypertension N = 3416^1^		Hypertension N = 2794^1^	
	Prevalence (95% CI)	P trend	Prevalence (95% CI)	P trend
Overall	38.8 (37.7–39.8)		31.5 (30.2–32.8)	
By geographic region				
Central	39.3 (37.6–41.0)	0.48	34.3 (32.6–36.0)	0.22
West	37.2 (35.0–39.4)		32.5 (30.3–34.8)	
West Nile	37.6 (35.5–39.7)		24.1 (22.0–26.3)	
North	34.8 (32.2–37.4)		22.0 (19.4–24.6)	
East	41.2 (38.9–43.4)		32.3 (30.2–34.4)	
By age category				
12–20 years^2^	37.6 (35.3–39.8)	0.05	12.8 (10.5–15.1)^2^	0.01
21–40 years	42.8 (41.2–44.5)		23.0 (21.5–24.4)	
41–60 years	34.7 (32.4–36.9)		49.8 (46.7–52.9)	
>60 years	30.3 (26.4–34.3)		56.2 (51.8–60.6)	

CI, confidence interval

^1^Pre-hypertension includes participants ≥12 years; hypertension restricted to those ≥18 years of age

^2^Hypertension restricted to participants 18 to 20 years of age

The overall age- and sex-adjusted prevalence of pre-hypertension was high (38.8%; 95% CI 37.7–39.8%), and it was similar across all regions (p-value for trend: 0.482). Nonetheless, it was highest in the East: 41.2% (95% CI 38.9 to 43.4%) and lowest the North 34.8% (95% CI 32.2 to 37.4%). The prevalence of pre-hypertension was highest among young adults and adolescents (p-value for trend: 0.048). Among those in the 21–40 years age category, it was 42.8% (95% CI 41.2–44.5%), and it was 37.6% (95% CI 35.3–39.8%) in the 12–20 years age category (**Table 4**).

### Predicted probability of BP phenotypes across age distribution

**Fig 2:**
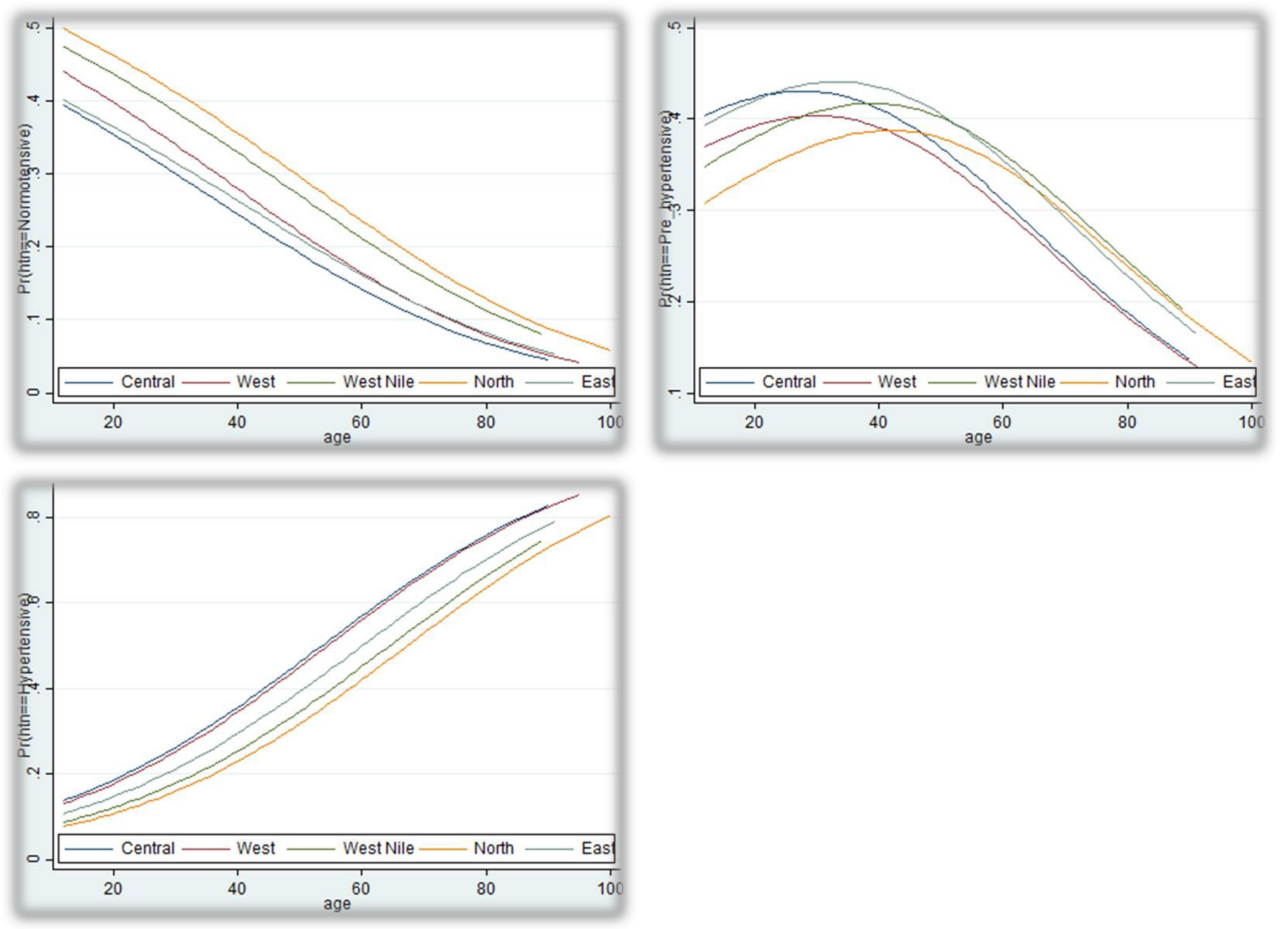
Predicted probabilities of being normotensive (left, upper: BP <120/80 mmHg), pre-hypertensive (right, upper: BP ≥120/80 but <140/90 mmHg), or hypertensive (left, lower: BP ≥140/90 mmHg) in five regions of Uganda according to age, years.

**Fig 2** illustrates predicted probabilities of BP statuses (normotensive, pre-hypertensive, and hypertensive) across age. The predicted probability of being pre-hypertensive increased gradually with age up until middle age (about 40 years old)–which coincided with an increase in the probability of being hypertensive–after which it decreased steadily with advancing age. There were substantial regional differences in the inflection point of the predicted probability of being pre-hypertensive–that is, the age at which the slope of the predicted probability became zero. This threshold occurred at an earlier age in the Central and the West and at a later age in West Nile, the East, and the North (**Fig 2B**).

## Discussion

In this nationally representative cross-sectional survey, the overall age- and sex-adjusted prevalence of hypertension among adults (≥18 years of age) in Uganda was 31.5%, and for pre-hypertension, it was 38.8%. According to region, the age- and sex-adjusted prevalence of hypertension was highest in the Central (34.3%), West (32.5%), and East (32.3%) Regions and lowest in the North (22.0%) and West Nile (24.1%) Regions. Ostensibly traditional demographic factors (age, gender, educational attainment, occupation, and monthly income) did not account for the lower prevalence of hypertension in the North and West Nile compared with the Central Region–suggesting that epidemiologic factors related to lifestyle and dietary changes may also play a role in the escalating burden of hypertension in Uganda. Further, pre-hypertension was highly prevalent among young adults (age ≤40 years) in all regions. Thus, to inform public health interventions targeted at high-risk groups, including young adults, future studies should delineate specific factors related to the epidemiologic transition and their potential role in conferring greater risk for hypertension in Uganda.

Our finding of a high prevalence of hypertension in Uganda is consistent with recent reports from several countries in sub-Saharan Africa [12,15–18], including prior reports from Uganda.^8^ For example, a study from Ethiopia found a high age-adjusted prevalence of hypertension among men (31.5%) and women (28.9%) in Addis Ababa in 2016 [15]. In another study from a rural community in South Africa, the prevalence of hypertension was 38.2% [16]. Similarly, a high prevalence of hypertension has been reported in many other countries in the region, including Tanzania, Ghana, and Nigeria [12,17–18]. Thus, our finding corroborate this body of evidence in Uganda and, taken together, they affirm the notion [18–22] of a region-wide epidemic of hypertension in sub-Saharan Africa, which is probably related to rapid urbanization in the region. Epidemiologic studies highlighting groups that are particularly at increased risk for hypertension in the region are therefore warranted to inform delivery of targeted interventions.

In Uganda, prior work has yielded conflicting evidence regarding the epidemiologic profile of individuals who are at increased risk for hypertension. While some studies suggest a disproportionately higher prevalence of hypertension in the Eastern [6] and Western [7] Regions of the country, a recent countrywide survey found no evidence in support of regional disparities in the prevalence of hypertension in Uganda [8]. Despite similar designs and analytic approaches, our finding departs from that of Guwatudde and colleagues [8] in that the prevalence of hypertension was significantly lower in the North and West Nile compared with the Central Region, which persisted after adjusting for demographic factors. Additional studies are warranted to determine reasons for this discrepancy.

Nonetheless, the geographic disparity in the demographic-adjusted prevalence of hypertension in our study gives credence to a multifactorial etiological model of hypertension in Uganda. For instance, the inflection point of the predicted probability of pre-hypertension (which was largely driven by a proportionate increase in the predicted probability of hypertension) occurred at lower age thresholds in low prevalent regions (North and West Nile) compared with high prevalent regions (Central and West)–although the slope for the predicted probability of hypertension across the age distribution was comparable in all regions. Therefore, efforts to mitigate risk for hypertension should consider multiple domains of factors, including demographic, environmental, behavioral, and genetic factors, that confer risk for hypertension in Uganda and other sub-Saharan African countries.

We noted some limitations. Because of the cross-sectional design of our study, we were not able to assess BP progression in participants over time. As such, the region-specific trajectory of BP status across age that we demonstrated in our study does not necessarily reflect patterns of BP progression among individuals in Uganda. Longitudinal assessments of changes in BP status among individuals will be required to elucidate potential regional differences in the rate of progression from pre-hypertensive BP to hypertension in Uganda. Despite this limitation, our study has important strengths. The large sample size generated sufficient degrees of freedom to detect regional differences in the prevalence of hypertension in Uganda, and the 3-stage countrywide cluster sampling strategy permitted enrollment of a nationally representative sample, which is generalizable to the Ugandan population.

In conclusion, Uganda has a very high burden of hypertension and pre-hypertension affecting individuals across the entire life course. The age- and sex-adjusted prevalence of hypertension were significantly lower in the less urbanized North and West Nile Regions suggesting factors related to lifestyle and dietary changes may play an important role. Future investigation should identify epidemiologic factors that confer greater risk for hypertension in the Central, Western, and Eastern Regions of Uganda.

## Supporting information

S1 File Detailed description of the protocol used for computing survey weights. (DOCX)
