# Supplementary material for: Geographic differences in the prevalence of hypertension in Uganda: Results of a national epidemiological study
Source: PLoS One. 2018 Aug 1;13(8):e0201001. doi: 10.1371/journal.pone.0201001 (PMC6070243; doi:10.1371/journal.pone.0201001)
Supplement: S1 File — (DOCX) [file pone.0201001.s001.docx]

**Supplemental File**

**Computing sampling weight for the Uganda National Asthma Survey**

We computed sampling weight using a 3-stage process:

1. We derived sampling probabilities for each of the three sampling levels using the formula n/N. Where “n” denotes the number of units sampled and “N” denotes the total number of units in the sampling frame. Table 1 details the number of units sampled at each level.

Table 1: number of sampling units per sampling level for the UNAS study

| **Sampling level** | **No. of units sampled** |
| --- | --- |
| District^1^ | 5 |
| Cluster |  |
| Kampala | 20 |
| Other districts | 10 |
| Households |  |
| Kampala | 20 |
| Other districts | 25 |
| ^1^Districts sampled include Kampala, Kiruhura, Pader, Maracha, and Iganga | |

1. Thereafter, we computed the overall sampling probability at the household level (primary sampling unit) by taking the product of the three sampling probabilities from the previous stage.
2. In the third stage, we computed the sampling weight as the reciprocal of the overall sampling probability from stage 2.
